# Adipose-derived MSC extracellular vesicles ameliorate sepsis by reprogramming macrophages via miR-21-5p targeting *PELI1*

**DOI:** 10.17305/bb.2025.11971

**Published:** 2025-10-27

**Authors:** Guannan Zhou, Jieqiong Song, Lizhen Xuan, Zhunyong Gu, Yimei Liu, Cheng Xu, Hongyu He

**Affiliations:** 1 Department of Intensive Care Unit, Zhongshan Hospital, Fudan University, Shanghai, China; 2 Department of Gynecology, The Obstetrics and Gynecology Hospital of Fudan University, Shanghai, China; 3 Department of Breast Surgery, Yangpu Hospital, School of Medicine, Tongji University, Shanghai, China

**Keywords:** ADMSCs, extracellular vesicles, EVs, sepsis, macrophages, IL-10, miR-21-5p

## Abstract

Sepsis is a common and life-threatening condition encountered in intensive care units (ICUs). Mesenchymal stromal cells (MSCs) and their small extracellular vesicles (EVs) have emerged as promising nanotherapeutics, particularly in the context of COVID-19. This study evaluates the efficacy and mechanisms of adipose-derived MSC EVs (ADMSC-EVs) in a lipopolysaccharide (LPS)-induced sepsis model. We quantified M2 macrophages and IL-10 in peripheral blood mononuclear cells from both septic patients and healthy donors. ADMSCs and their EVs were isolated, and EVs were administered to LPS-challenged mice. Macrophage phenotypes in lung tissue were analyzed using flow cytometry and immunofluorescence. The biodistribution of EVs was traced with PKH67 green fluorescent cell linker dye (PKH-67), and the signaling pathways involved in macrophage reprogramming were examined. ADMSC-EVs efficiently entered macrophages, promoted M2 polarization, suppressed inflammation, and improved survival rates in septic mice. Biodistribution studies demonstrated widespread organ accumulation, with notable localization in the lungs, liver, and kidneys. Mechanistically, the EV cargo miR-21-5p targeted Pellino E3 ubiquitin protein ligase 1 (*PELI1*), driving M2 polarization *in vivo*, which was accompanied by increased IL-10 levels. These findings position ADMSC-EVs as a viable cell-free therapeutic approach for mitigating LPS-induced sepsis through the delivery of miR-21-5p to *PELI1*, thereby supporting further development of EV-based immunomodulatory strategies for sepsis management.

## Introduction

Sepsis is a systemic infection syndrome that triggers immune dysregulation and can lead to multiple organ dysfunction [1, 2]. Increasing evidence suggests that sepsis is a leading cause of mortality in intensive care units (ICUs) across the United States and Europe [3, 4]. The pathophysiology of sepsis involves a complex interplay among the immune system, microbiome, and host tissues. Consequently, regulating the inflammatory response remains a significant challenge in sepsis management. While inflammation is essential for controlling infection, excessive or prolonged activation can result in tissue damage, organ dysfunction, and ultimately, sepsis-induced mortality. Current standard treatments for sepsis include antibiotics, fluid resuscitation, corticosteroids, and vasopressors; however, these therapies primarily focus on symptom management and infection control rather than addressing the underlying immune dysfunction. Early and broad-spectrum antibiotic therapy is the cornerstone of sepsis treatment, aimed at targeting the underlying infection. Nevertheless, the emergence of antimicrobial resistance poses a substantial challenge to effective sepsis management [5, 6]. Corticosteroids are sometimes employed to counteract the inflammatory response in severe sepsis, yet their use remains controversial as they can suppress the immune system, increase the risk of secondary infections, and prolong ICU stays [7]. New approaches that involve immune modulation and tissue repair regulation are recognized as promising strategies. Therapeutics aimed at restoring macrophage function, promoting immune tolerance, and modulating the inflammatory response may serve as valuable adjuncts to existing sepsis therapies.

Mesenchymal stem cells (MSCs) exhibit potent immunomodulatory effects that can mitigate sepsis severity. MSCs, which are multipotent stromal cells capable of differentiating into various cell types, can modulate immune responses, promote tissue regeneration, and accelerate healing processes. Mechanistically, MSCs can reduce pro-inflammatory cytokines while simultaneously increasing anti-inflammatory cytokines [8–10]. Recently, adipose-derived MSCs (ADMSCs) have emerged as promising candidates for clinical application. Research has demonstrated that ADMSCs can alleviate liver injury [11], reduce systemic inflammation [12], promote tissue regeneration, and enhance survival outcomes in septic models.

Extracellular vesicles (EVs), as nanocarriers derived from various cell types, facilitate intercellular communication by delivering bioactive cargo to recipient cells [13–16]. One of the primary mechanisms through which ADMSCs exert their therapeutic effects is via the secretion of EVs, which are nanosized membrane-bound particles that carry an array of bioactive molecules, including proteins [17], lipids, and RNA [18, 19]. EVs derived from ADMSCs demonstrate anti-inflammatory, immunomodulatory, and regenerative properties, making them promising candidates for sepsis therapy. Among their bioactive cargo, microRNAs (miRNAs) have emerged as key regulators of gene expression. These small, non-coding RNAs fine-tune cellular responses and influence a broad range of biological processes, including immune regulation [20], inflammation [21], and tissue repair. miRNAs contained within ADMSC-derived EVs have been found to play a crucial role in modulating macrophage polarization and function, which is vital in the context of sepsis. Increasing evidence indicates that miR-21-5p can inhibit lipopolysaccharide (LPS)-induced inflammatory injuries [22, 23], while a decrease in exosomal miR-21-5p correlates with the progression of sepsis in polytraumatized patients [24]. Additionally, one study showed that exosomal microRNA-21-5p derived from endothelial progenitor cells alleviates sepsis-induced acute kidney injury by inhibiting *RUNX1* expression [25]. Our previous study revealed that high levels of miR-21-5p are present in MSCs and MSC-derived Evs [13]. Therefore, understanding the role of ADMSC-derived EV miRNAs in sepsis treatment is crucial for developing effective cell-free therapeutic strategies that leverage the potential of stem cell-derived vesicles. This leads us to hypothesize that adipose-derived MSC EVs (ADMSC-EVs) enriched with specific miRNAs could similarly alleviate related disorders. In this study, we investigate the role of ADMSC-EVs enriched with miR-21-5p in sepsis suppression and delineate the underlying mechanisms involved. Understanding the molecular mechanisms underlying EV-mediated effects in sepsis could pave the way for new, targeted treatments for this devastating condition.

## Materials and methods

### Ethics statement

This study was conducted following approval from the Ethical Committee of Zhongshan Hospital, Fudan University (approval number B2022-107R, 2022–03), with informed consent obtained from all patients. All personal data collected during the study will be anonymized prior to analysis, ensuring that no identifiable information is retained. Additionally, any biological samples provided may be stored in a certified biobank for future ethically approved research. All animal procedures were performed in accordance with protocols approved by the Institutional Animal Care and Use Committee at Fudan University (SYXK2020-0032).

### Clinical samples

Patients with sepsis (*n* ═ 10) and non-sepsis donors (*n* ═ 10) were enrolled to collect fresh peripheral blood samples at Zhongshan Hospital, Fudan University, from 2020–2023. There were no significant differences in demographic characteristics, including age, sex, ethnicity, underlying health conditions, and infection types, between the sepsis and non-sepsis donor groups. Peripheral blood mononuclear cells (PBMCs) were isolated using the EasySep kit (STEMCELL Technologies, Canada).

### Isolation of peripheral blood mononuclear cells and analysis

PBMCs were collected from both septic patients and non-sepsis donors. Typically, 10 mL of peripheral blood was collected in tubes containing EDTA anticoagulant, and isolation was completed within 2 h. 10 mL of PBS was added to the peripheral blood in a 50 mL falcon tube, which was subsequently layered onto 20 mL of Lymphoprep (#07861, STEMCELL Technologies, Canada) in a new 50 mL falcon tube. The tubes were centrifuged at 600 g for 20 min, and the PBMC layer (the white layer in the middle) was collected into a new 50 mL falcon tube and washed twice with PBS. The PBMCs were then cultured in RPMI-1640 (containing 10% FBS and 1% penicillin and streptomycin) or frozen at –80 ^∘^C.

For quantitative PCR (qPCR), total RNA was isolated and purified using the RNApure extraction kit (BioTeke Corporation, Beijing, China) according to the manufacturer’s instructions. Cell lysates were extracted using Trizol reagent (Invitrogen, United States) to determine mRNA levels.

For flow cytometry, cells were first incubated with 2% FBS in PBS to block non-specific binding sites, followed by surface staining with fluorochrome-conjugated antibodies against specific markers for 30 min at 4 ^∘^C in the dark. Intracellular staining for IL-10 and IL-6 was conducted next. Cells were fixed and permeabilized using Fixation/Permeabilization solution (BD Biosciences) before being stained with corresponding intracellular antibodies. Samples were analyzed using a CytoFLEX S Flow Cytometer (Beckman Coulter).

### ADMSCs production

ADMSCs were collected from C57BL/6 mice to produce ADMSC-derived EVs (ADMSCs-EVs). Typically, six 6-week-old C57BL/6 mice (three males and three females) were used for each production batch. The animals were randomly assigned to groups to ensure unbiased allocation. Adipose tissue samples were collected from the inguinal fat pad of the mice, cut into small pieces, and digested using a 0.1% type I collagenase solution (Gibco, Grand Island, NY, USA) for 30 min. The digestion was halted with 10 mL of DMEM (containing 20% FBS). After filtering through a 70 µm nylon mesh twice, the suspension was centrifuged at 1500 g for 5 min to isolate the cells. The cells were resuspended and incubated in DMEM (containing 10% FBS and 100 U/mL of penicillin and streptomycin) and cultured together.

### Isolation and purification of ADMSCs-EVs

Initially, EV-free FBS was prepared for cell culture by centrifuging FBS for 12 h at 120,000 g to remove EVs. The isolated ADMSCs were cultured in DMEM medium supplemented with EV-free FBS. The conditioned medium was subsequently collected and centrifuged at 2000 g for 30 min to remove cell debris and apoptotic bodies, followed by tangential flow filtration (TFF), as reported in previous studies [26–29]. The ADMSC-EVs were resuspended in cold PBS and filtered through a 0.22 µm filter. Characterization assays included transmission electron microscopy (TEM), nanoparticle tracking analysis (NTA), and Western blotting. In the TEM assay, diluted EVs were loaded onto carbon-coated copper electron microscope grids for 1 min and negatively stained with phosphotungstic acid for 10 min. Images were observed using a TEM (FEI Tecnai G2 Spirit Twin, Philips, NL). In the NTA assay, EVs were dissolved in 1000 µL of PBS, and particle size distribution was analyzed using a NanoSight nanoparticle tracking analyzer (NTA; Malvern, U.K.).

### Plasmid production and transfection

The Plex-CD63 (Addgene, #168220) and Plex-GFP (Addgene, #162032) plasmids were acquired from Addgene. The “Plex-CD63-GFP” plasmid was constructed by cloning the purchased plasmids using restriction enzymes.

Prior to transfection, ADMSCs were seeded in T225 flasks with 30 mL of culture medium and incubated at 37 ^∘^C with 5% CO_2_ for 12 h. For each T225 flask, 25 µg of plasmid DNA was mixed with 3 mL of Opti-MEM (Thermo Fisher Scientific) and incubated for 5 min. Concurrently, 50 µL of PEI4000 (40816ES02, Yeasen Biotechnology) was mixed with Opti-MEM for 5 min. The two solutions were combined and incubated for an additional 20 min before being added to the flasks. To facilitate EV production, the culture medium was replaced with EV-free DMEM (containing 10% EV-free FBS) for 48 h. TFF was employed to purify the EVs, removing residual plasmids and PEI. The supernatant was subsequently collected for EV isolation.

### Enzyme-linked immunosorbent assay (ELISA)

THP-1 cells cultured in 24-well plates were treated with phorbol 12-myristate 13-acetate (PMA) to induce the M0 phenotype. Subsequently, PBS (C0221A, Beyotime Biotechnology), THP1-derived EVs, and ADMSC-derived EVs were added to the M0 cells and incubated for 48 h. Human IL-6 (ab178013) and IL-10 (ab185986) ELISA kits were utilized to quantify the expression levels of these cytokines according to the manufacturer’s protocols. In the animal study, plasma samples were analyzed using ELISA kits (ab222503) and a mouse TNF-alpha ELISA kit (ab208348), following the manufacturer’s specifications.

### Immunofluorescence and confocal microscopy

To characterize ADMSCs, immunofluorescence assays were conducted. Cells cultured on round coverslips were fixed in 4% paraformaldehyde (PFA) for 20 min and permeabilized with 0.3% Triton X-100 for 20 min. Blocking was performed using a blocking buffer (Solarbio). Primary and secondary antibodies were diluted in Immunofluorescent Staining Dilution Buffer (Beyotime Biotechnology). The cells were incubated with primary and secondary antibodies according to the manufacturer’s instructions. Anti-CD90 (ab307736), Anti-CD105 (ab221675), and Alexa Fluor 488 Goat anti-Rabbit secondary antibody (A0423, Beyotime Biotechnology) were employed. Images were captured using a confocal microscope (Nikon, Japan).

### EV labeling

EVs were labeled using PKH67 (Sigma-Aldrich PKH67GL) according to the manufacturer’s instructions and previous studies [30, 31]. Briefly, EVs in PBS were combined with 0.5 mL of Diluent C, and 3 µL of PKH67 was added to 0.5 mL of Diluent C; the two mixtures were incubated at room temperature for 20 min. Subsequently, 20 mL of EV-free FBS was added to prevent excessive labeling, followed by ultracentrifugation at 125,000 × g for 1 h to remove residual dye. The recipient cells were treated with the PKH67-labeled EVs for 4 h. After treatment, cells were washed twice with PBS, fixed with 4% PFA for 10 min, and stained with mCherry-Actin-Tracker and DAPI. Samples were observed and captured using a confocal microscope (Nikon, Japan).

### Animal assay

For each batch of ADMSC production, six C57BL/6 mice (three male and three female, all 6 weeks old) were utilized to establish an LPS-mediated sepsis model. Mice were randomly assigned to one of three groups (*N* ═ 5 mice/group: 2 male and 3 female): LPS group (treated with LPS only), LPS+PBS group (treated with LPS followed by PBS intravenous injection), and ADMSC-EVs group (treated with LPS followed by intravenous injection of ADMSC-derived EVs labeled with PKH67). Randomization was performed to ensure unbiased allocation of subjects. Mice were placed in an induction chamber filled with isoflurane (2.5% induction concentration) for approximately 40 s. The chamber was then closed until the mice were fully anesthetized (approximately 1.5 min). An anesthetized mouse would remain in a lateral position without returning to an upright posture. A total of 4 mg/kg of LPS was administered intraperitoneally to induce sepsis. Subsequently, 10^11^ particles of ADMSC-EVs were injected intravenously at 0, 6, and 12 h post-LPS treatment. As a control, an equivalent volume of PBS was injected via the same route. Animals were monitored every 6 h during the first 48 h post-procedure and then every 12 h for 6 days. Ultimately, all mice were euthanized via intraperitoneal injection of pentobarbital sodium at a dosage of 150 mg/kg. The animal experiments were conducted in accordance with the guidelines for the care and use of laboratory animals (SYXK2020-0032). Group assignments were blinded during the study to minimize bias. Euthanasia was performed when animals exhibited signs of weight loss exceeding 20%. All procedures were conducted following institutional ethical guidelines and under veterinary oversight to minimize animal suffering.

### Phenotyping THP-1 cells

THP-1 is a human leukemia monocytic cell line widely used to investigate monocyte/macrophage functions [32, 33]. The cells were treated with 100 nM PMA (Sigma-Aldrich) for 24 h. To induce M2 macrophage polarization, the differentiation medium was replaced with RPMI-1640 supplemented with 10% FBS, along with the addition of polarizing agents: IL-4 (20 ng/mL) and IL-13 (20 ng/mL), which are key cytokines that drive M2 polarization through activation of the STAT6 pathway, promoting the anti-inflammatory M2 phenotype. The polarization of the induced cells was subsequently assessed using flow cytometry.

### Flow cytometry

We cultured ADMSCs and assessed the expression of surface biomarkers at the third passage using FITC-CD90 (ab25672) and FITC-CD105 (ab314950) antibodies for phenotyping via flow cytometry. For the *in vivo* assay, half of the lung tissues underwent immunofluorescence analysis, while the other half was lysed to create a single-cell suspension for flow cytometry. The macrophages were stained using PE-Cy7 Mouse Anti-Human CD68 (BD Pharmingen, 565595), FITC Mouse Anti-Human CD206 (BD Pharmingen, 551135), and FITC Rat Anti-Mouse IL-10 (BD Pharmingen, 554466). In the THP-1 phenotyping assay, the induced cells were stained with CD68 and CD206 antibodies. To evaluate the role of ADMSC-derived EVs (ADMSC-EVsGFP) in septic mice, we selected PE Rat Anti-Mouse F4/80 (BD Pharmingen, T45-2342) and Alexa Fluor 647 Rat Anti-Mouse CD206 (BD Pharmingen, MR5D3) antibodies. All primary conjugated antibody incubations were performed at room temperature.

### Western blot assay

Total protein was extracted using RIPA buffer (P0013C, Beyotime Biotechnology) supplemented with 1 mM phenylmethylsulfonyl fluoride (PMSF). Protein concentrations were determined using the Bradford protein quantification assay kit. A total of 20 µg of EVs or cell lysates were resuspended in 5× SDS loading buffer and incubated at 100 ^∘^C for 20 min. The supernatant was separated by 10% SDS-polyacrylamide gel (Beyotime Biotechnology) and transferred to PVDF membranes (Millipore), which were then blocked with blocking buffer (Beyotime Biotechnology) for 1 h. Primary antibodies were incubated overnight at 4 ^∘^C, followed by a 1-h incubation with secondary antibodies. An ECL kit (Beyotime Biotechnology) was used for band detection. EVs were detected using anti-CD63 (ab134045), anti-Alix (ab275377), and anti-Tsg101 (ab133586), which have been previously validated in studies [34–37]. Cell samples were analyzed using anti-IL10 (ab52909) and anti-Tubulin (ab6160).

**Table 1:** qRT-PCR primers used for human and mouse genes

**Gene name sequence (5′-3′)**	**qRT-PCR primers**
*CD163* (human)	F: TTTGTCAACTTGAGTCCCTTCAC
	R: TCCCGCTACACTTGTTTTCAC
*CD163* (mouse)	F: TTTGTCAACTTGAGTCCCTTCAC
	R: TCCCGCTACACTTGTTTTCAC
*IL10* (human)	F: GACTTTAAGGGTTACCTGGGTTG
	R: TCACATGCGCCTTGATGTCTG
*IL10* (mouse)	F: GACTTTAAGGGTTACCTGGGTTG
	R: TCACATGCGCCTTGATGTCTG
*ARG1* (human)	F: TTGGGTGGATGCTCACACTG
	R: GTACACGATGTCTTTGGCAGA
*Arg-1* (mouse)	F: TTGGGTGGATGCTCACACTG
	R: GTACACGATGTCTTTGGCAGA
*GAPDH* (human)	F: CGGAGTCAACGGATTTGGTCGTAT
	R: AGCCTTCTCCATGGTGGTGAAGAC
*Gapdh* (mouse)	F: CGGAGTCAACGGATTTGGTCGTAT
	R: AGCCTTCTCCATGGTGGTGAAGAC
miR21-5p (human)	F: CCCCCTAGCTTATCAGACTGATG
	R: CCAGTGCAGGGTCCGAGGT
miR21-5p (mouse)	F: CCCCCTAGCTTATCAGACTGATG
	R: CCAGTGCAGGGTCCGAGGT
U6 (human)	F: CGCTTCACGAATTTGCGTGTCAT
	R: GCTTCGGCAGCACATATACTAAAAT
U6 (mouse)	F: CGCTTCACGAATTTGCGTGTCAT
	R: GCTTCGGCAGCACATATACTAAAAT
*CD68* (human)	F: AGGAGACACAGGAAATGGAG
	R: ATGGATGCTGGAGTGATGAA
*CD68* (mouse)	F: CCGGAAGAAAGGAGATAGGAG
	R: CCTGAGGACTCCTTCCATG
*IL6* (human)	F: GAAAGCAGCCAGAGTCATTC
	R: CAAGGAGGAGACTTGCAGAGA
*IL6* (mouse)	F: CAAAGGAGGAGACTTGCAGAGA
	R: CAAGGAGGAGACTTGCAGAGA

Abbreviations: *Arg-1*: Arginase 1; *GAPDH*: Glyceraldehyde-3-phosphate dehydrogenase; F: Forward primer; R: Reverse primer.

### Quantitative real-time PCR

For the measurement of miRNAs in EVs, RNA extraction was performed on isolated EVs using the miRNeasy Micro Kit (Qiagen).

Gene expression levels of *CD163*, *Arg1*, and *IL-10* were assessed at the mRNA level. Total RNA was isolated and purified using the RNApure extraction kit (BioTeke Corporation, Beijing, China) following the manufacturer’s instructions, with cells treated with PBS serving as controls. Subsequently, cell lysates were extracted using Trizol reagent (Invitrogen, United States) to determine the mRNA levels of *CD163*, *Arg1*, and *IL-10*. Real-time PCR (RT-PCR) was conducted using the TB Green premix Ex Taq II (TaKaRa Biotechnology, Dalian, China) in the CFX96 Real-Time PCR Detection System (Bio-Rad, United States). The levels of miR-21-5p were normalized against U6 snRNA (MQP-0202, RiboBio, Guangzhou, China), and results were calculated using the 2^-ΔΔCt^ method. The relative expressions of the genes were calculated with GAPDH as an internal reference. Specific primers are listed in Table 1.

### Luciferase reporter assays

Potential targets of miR-21-5p were predicted using TargetScan (http://www.targetscan.org) and miRanda (http://miranda.org). Fragments of the PELI1 3’ UTR containing either the wild-type (WT) or mutant (Mut) predicted miR-21-5p binding site were subcloned into the pmirGLO vector (RiboBio). Subsequently, cells were cultured and seeded in 24-well plates for further analysis. MiR-21-5p mimics (5′-CAACACCAGUCGAUGGGCUGU-3′) or control sequences (5′-UUCUCCGAACGUGUCACGUTT-3′) were co-transfected with pmirGLO-PELI1 WT or pmirGLO-PELI1 Mut constructs. MiR-21-5p mimic (B02003), mimic control (MC, B04002), inhibitor (B03001), and inhibitor control (B04003) were synthesized by Genepharma (Shanghai, China). After 48 h of transfection, cells were harvested and lysed. Firefly luciferase and Renilla luciferase substrates were added, and luciferase activity was measured using the Dual-Glo Luciferase Reporter Assay System (Promega), with results analyzed according to the manufacturer’s instructions.

### Ethical statement

This study was conducted with approval from the Ethical Committee of Zhongshan Hospital, Fudan University, under approval number B2022-107R (2022-03), with informed consent obtained from all patients. All animal procedures adhered to protocols approved by the Institutional Animal Care and Use Committee at Fudan University (SYXK2020-0032). The study complied with the ethical standards set forth in the Declaration of Helsinki.

### Statistical analysis

Data are presented as mean ± SEM. Statistical differences were determined using the unpaired two-tailed Student’s *t*-test or Mann–Whitney test, as appropriate. Kaplan–Meier survival plots and log-rank tests were employed to compare survival outcomes among treatment groups. Significance levels were denoted as *P <* 0.05, *P* < 0.01, and *P* < 0.001, with statistical significance defined as *P* < 0.05.

## Results

### Altered M2 macrophage proportion and *IL-10* expression in peripheral blood of sepsis patients

**Figure 1.**
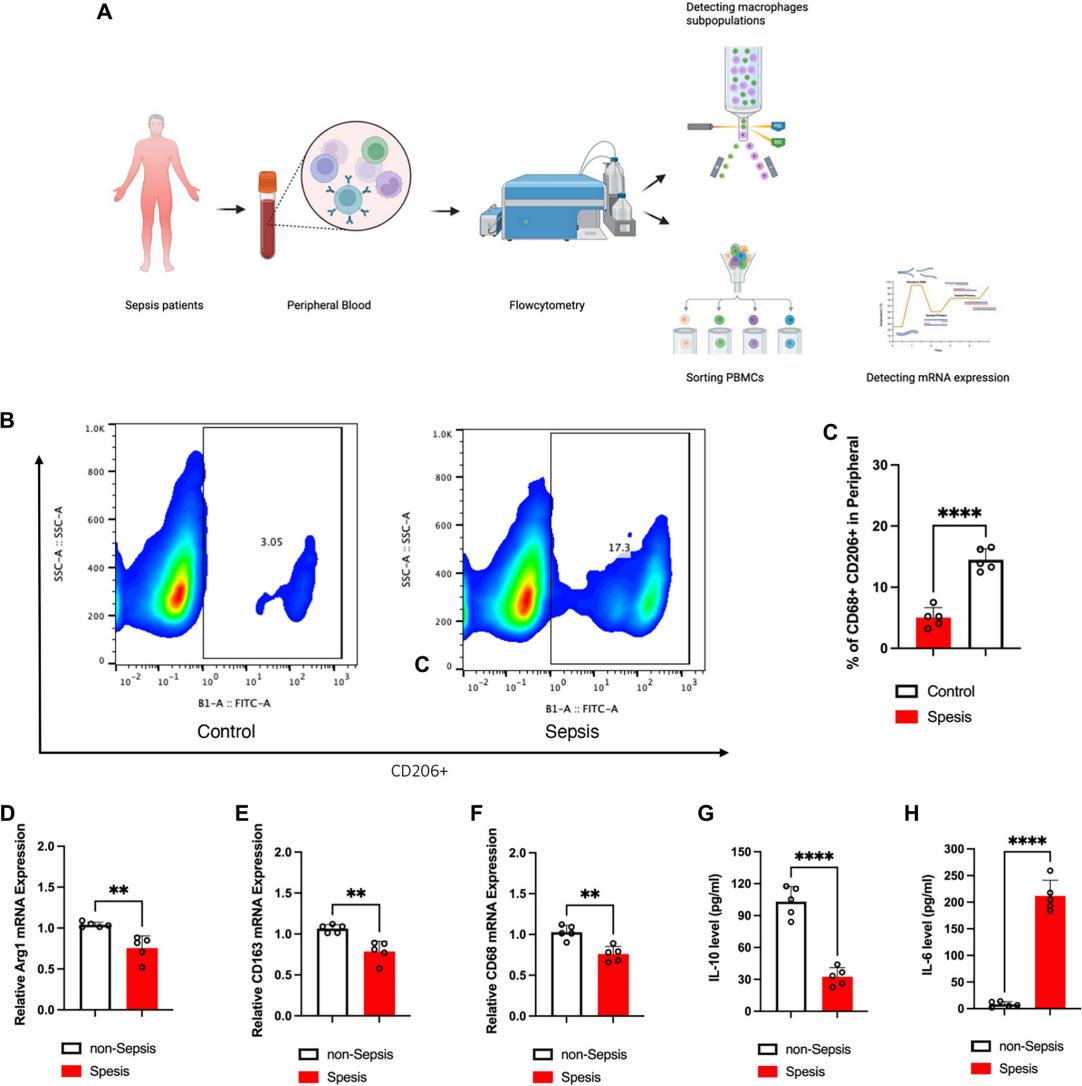
**Macrophage phenotype subpopulations in serum with and without sepsis.** (A) Flowchart for the detection of macrophage phenotype subpopulations in serum from patients with and without sepsis; (B) Representative image showing the percentage of CD68+CD206+ cells in serum from patients with and without sepsis, as determined by flow cytometry; (C) Quantitative analysis of the percentage of CD68+CD206+ cells in serum from patients with sepsis (*n* ═ 10) compared to those without sepsis (*n* ═ 10); (D) mRNA expression levels of *Arg1* in PBMCs from patients with sepsis (*n* ═ 10) vs those without sepsis (*n* ═ 10); (E) mRNA expression levels of *CD163* in PBMCs from patients with sepsis (*n* ═ 10) and without sepsis (*n* ═ 10); (F) mRNA expression levels of *CD68* in PBMCs from patients with sepsis (*n* ═ 10) and without sepsis (*n* ═ 10); (G) Cytokine expression levels of IL-10 in serum from septic patients (*n* ═ 10) compared to non-septic patients (*n* ═ 10); (H) Cytokine expression levels of IL-6 in serum from septic patients (*n* ═ 10) vs non-septic patients (*n* ═ 10). Abbreviations: *Arg1*: Arginase 1; PBMCs: Peripheral blood mononuclear cells.

To investigate the role of macrophages in sepsis, we analyzed the CD68+CD206+ macrophage subpopulation (M2) in the PBMCs of septic patients (Figure 1A). The results, illustrated in Figure 1B and 1C, indicated a significantly reduced proportion of CD68+CD206+ M2 macrophages in septic patients compared to non-septic individuals. Furthermore, the mRNA expression levels of *Arg1*, *CD163*, and *CD68* in PBMCs from septic patients were lower than those in non-septic patients. Additionally, serum cytokine levels revealed a decrease in IL-10 and an increase in IL-6 among septic patients. Collectively, these findings suggest that both M2 macrophage proportions and *IL-10* expression are diminished in sepsis, underscoring their critical roles in mitigating the progression of the condition.

### Characterization of ADMSCs and ADMSCs-EVs and their potential in sepsis therapy

**Figure 2.**
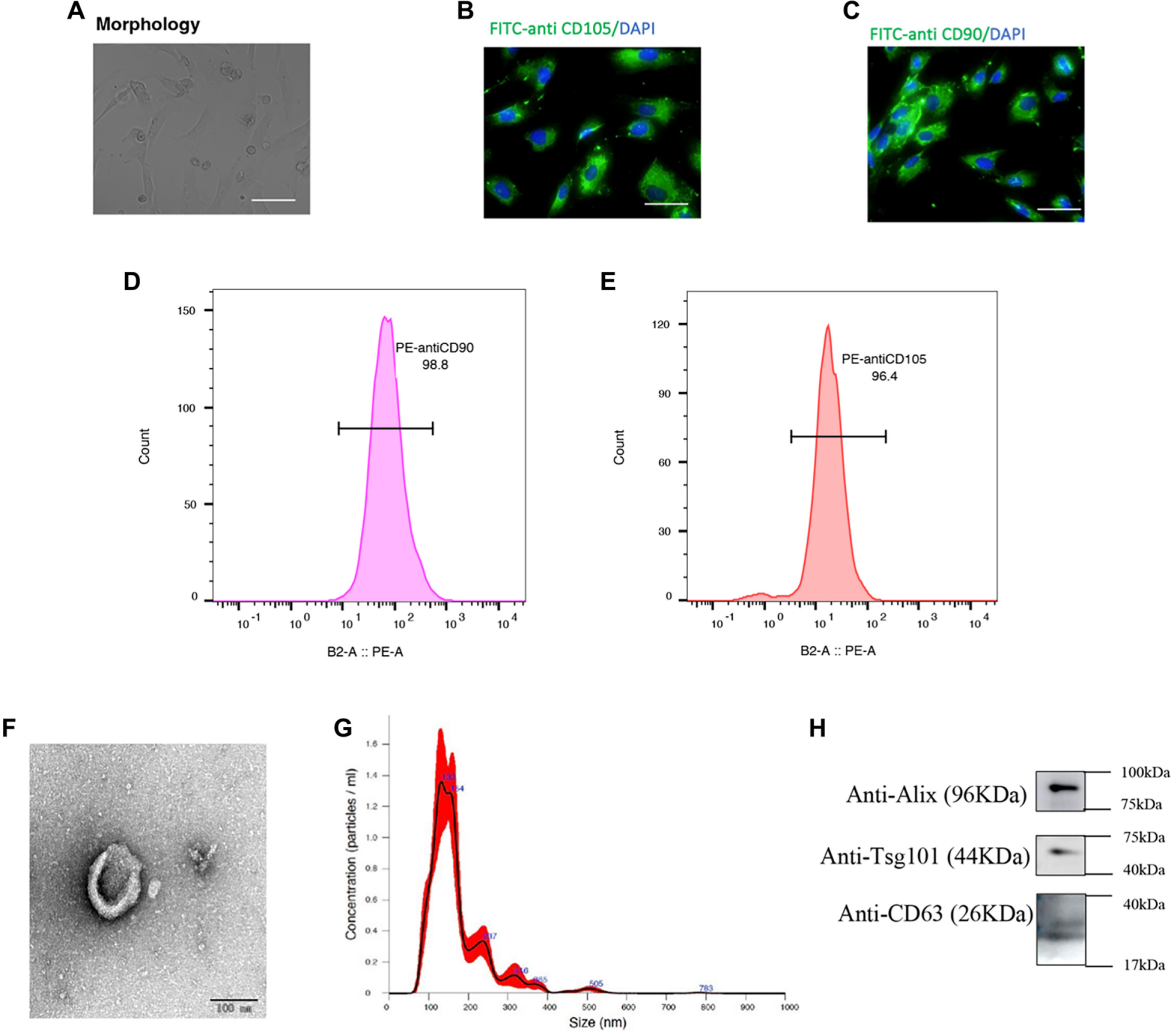
**Isolation and identification of ADMSCs and ADMSC-EVs.** (A) The morphological characteristics of primary ADMSCs were examined using a microscope; (B) The surface marker CD105 of ADMSCs was detected using fluorescence microscopy; (C) The surface marker CD90 of ADMSCs was also detected via fluorescence microscopy; (D) The expression of CD105 in ADMSCs is shown, with blue indicating the isotype control and red indicating CD90 positivity; (E) The expression of CD90 in ADMSCs is depicted, with red representing the isotype control and blue indicating CD90 positivity; (F) The size distribution of ADMSC-EVs was analyzed using NTA; (G) The surface markers (CD63, Alix, and Tsg101) of ADMSC-EVs were evaluated by western blot analysis; (H) The morphological characteristics of ADMSC-EVs were observed using a transmission electron microscope. Abbreviations: ADMSCs: Adipose-derived mesenchymal stem cells; ADMSC-EVs: Adipose-derived mesenchymal stem cell-derived extracellular vesicles; NTA: Nanoparticle tracking analysis; Alix: ALG-2-interacting protein X.

This study aimed to assess the therapeutic potential of ADMSCs and ADMSCs-EVs in alleviating sepsis. We conducted a comprehensive characterization process, including adherence ability, surface marker expression, and differentiation potential. As shown in Figure 2A, ADMSCs displayed a spindle-like morphology and strong adherence. Immunofluorescence (Figure 2B and 2C) and flow cytometry analyses (Figure 2D and 2E) confirmed the expression of surface markers CD105 and CD90, consistent with established characteristics of MSCs. For EV isolation and characterization, ADMSCs-EVs were obtained using TFF, as reported in previous studies [26]. Characterization of ADMSCs-EVs was performed using NTA and TEM. Particle diameters were predominantly in the 30 to 500 nm range (Figure 2F). Additionally, marker proteins such as CD63, Alix, and Tsg101 were detected on ADMSCs-EVs (Figure 2G and Figure S1). TEM imaging revealed cup-shaped structures (Figure 2H). The secretion efficiency of ADMSCs was also analyzed via NTA (Figure S2). In summary, we successfully isolated ADMSCs and their EVs.

### ADMSC-EVs induce M2 polarization *in vitro*

**Figure 3.**
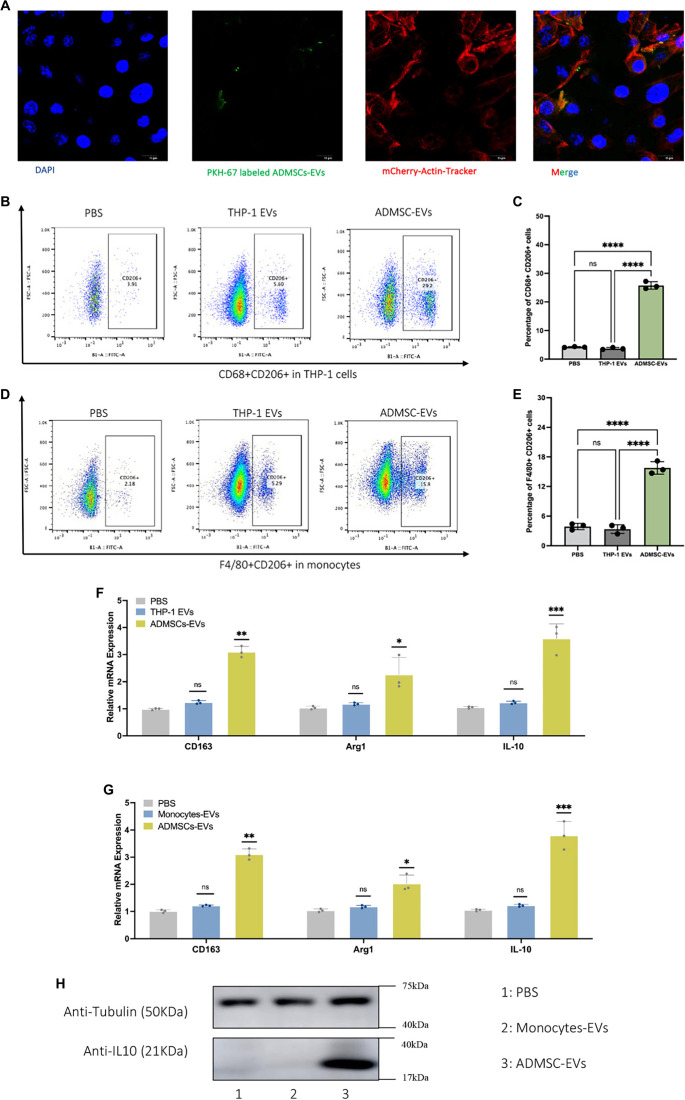
**ADMSC-EVs induce macrophages polarization into M2 phenotype *in vitro* and *ex vivo.*** (A) Uptake of ADMSC-EVs by THP-1 cells, as indicated by PKH-67 labeling and mCherry-Actin Tracker; (B) THP-1 cells treated with ADMSC-EVs exhibited a significantly higher percentage of CD68+CD206+ cells compared to those treated with PBS or THP-1 EVs; (C) Quantitative analysis of the percentage of CD68+CD206+ cells following various treatments; (D) Primary monocytes treated with ADMSC-EVs showed an increased percentage of CD68+CD206+ cells compared to those treated with PBS or THP-1 EVs; (E) Quantitative analysis of the percentage of CD68+CD206+ cells after different treatments; (F) mRNA expression levels of *CD163*, *Arg1*, and *IL-10* in THP-1 cells treated with PBS, THP-1 EVs, and ADMSC-EVs; (G) mRNA expression levels of *CD163*, *Arg1*, and *IL-10* in primary monocytes treated with PBS, THP-1 EVs, and ADMSC-EVs; (H) Protein expression levels of IL-10 in primary monocytes treated with PBS, Monocyte-EVs, and ADMSC-EVs. Abbreviations: ADMSC-EVs: Adipose-derived mesenchymal stem cell-derived extracellular vesicles; PKH-67: Lipophilic fluorescent membrane dye; PBS: Phosphate-buffered saline; EVs: Extracellular vesicles; Arg1: Arginase 1.

We evaluated the uptake of ADMSC-EVs and their role in macrophage reprogramming and polarization. As shown in Figure S3, THP-1 cells were treated with PKH-67 labeled ADMSCs-EVs for 72 h. The labeled ADMSCs-EVs effectively entered the mCherry-Actin Tracker labeled THP-1 (phenotyping as Figure S4) cells (nuclei labeled with DAPI) (Figure 3A). As depicted in Figure 3B and 3C (Figure S5), THP-1 cells treated with ADMSC-EVs exhibited significantly higher levels of CD206 expression compared to those treated with PBS or THP-1-EVs. Similar results were observed in primary mouse monocytes, where ADMSC-EVs promoted a higher percentage of the F4/80+CD206+ macrophage subpopulation compared to PBS or monocyte-derived EVs (Figure 3D and 3E and Figure S6). Moreover, ADMSC-EVs significantly increased the mRNA expression levels of *CD163*, *Arg1*, and *IL-10* in both THP-1 cells (Figure 3F) and primary monocytes (Figure 3G) compared to controls. Additionally, the protein expression level of IL-10 in ADMSC-EVs treated monocytes was higher than in the PBS and monocyte-EVs groups (Figure 3H and Figures S7 and S11). These results collectively indicate that ADMSC-EVs effectively promote macrophage polarization in both THP-1 cell lines and primary mouse monocytes.

### ADMSC-EVs mitigate LPS-mediated sepsis by inducing macrophages polarization in mice

**Figure 4.**
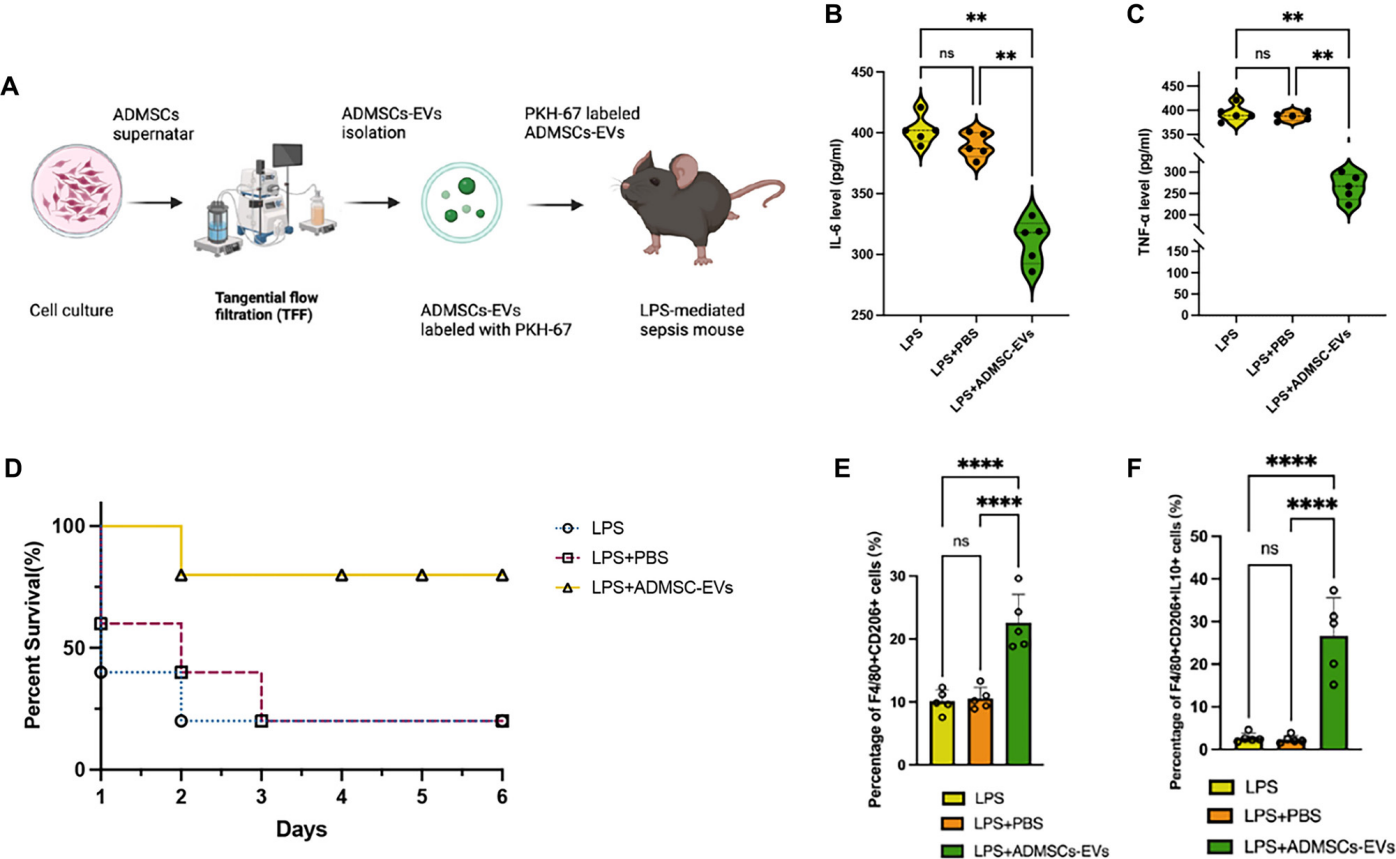
**ADMSC-EVs alleviate LPS-induced sepsis.** (A) Flowchart illustrating the isolation and injection of ADMSC-EVs into LPS-induced septic mice; (B) The expression levels of IL-6 in LPS-mediated septic mice, treated with no intervention, PBS, or ADMSC-EVs, were measured using ELISA; (C) The expression levels of TNF-α in LPS-mediated septic mice, treated with no intervention, PBS, or ADMSC-EVs, were assessed via ELISA; (D) The survival rates of mice over a 6-day period (LPS group: *n* ═ 5; LPS + PBS group: *n* ═ 5; LPS + ADMSC-EVs group: *n* ═ 5); (E) The percentage of F4/80+ CD206+ cells among PBMCs was analyzed following treatment with ADMSC-EVs, PBS, or no treatment for 12 h via flow cytometry; (F) The percentage of F4/80+ CD206+ IL-10+ cells among PBMCs was evaluated after treatment with ADMSC-EVs, PBS, or no treatment for 12 h via flow cytometry. Abbreviations: ADMSC-EVs: Adipose-derived mesenchymal stem cell-derived extracellular vesicles; LPS: Lipopolysaccharide; TNF-α: Tumor necrosis factor alpha; PBMCs: Peripheral blood mononuclear cells; PBS: Phosphate-buffered saline; ELISA: Enzyme-linked immunosorbent assay.

To evaluate the protective effects of ADMSC-EVs against endotoxic shock in an LPS-induced sepsis model, mice received LPS followed by ADMSC-EV treatment (Figure 4A). Figure 4B and 4C demonstrates that IL-6 and TNF-α levels in ADMSC-EVs treated LPS-induced septic mice were significantly lower than in control groups. Notably, mortality rates were substantially reduced in the ADMSC-EV group compared to control groups, with an 80% mortality rate due to LPS-induced sepsis (Figure 4D). Flow cytometry analyses revealed an increased percentage of F4/80+CD206+ macrophages and F4/80+CD206+IL-10+ macrophages in ADMSC-EVs treated LPS-mediated septic mice (Figure 4E and 4F).

### ADMSC-EVs reduce the inflammatory by increasing *IL-10* expression in M2 macrophages *in vivo*

**Figure 5.**
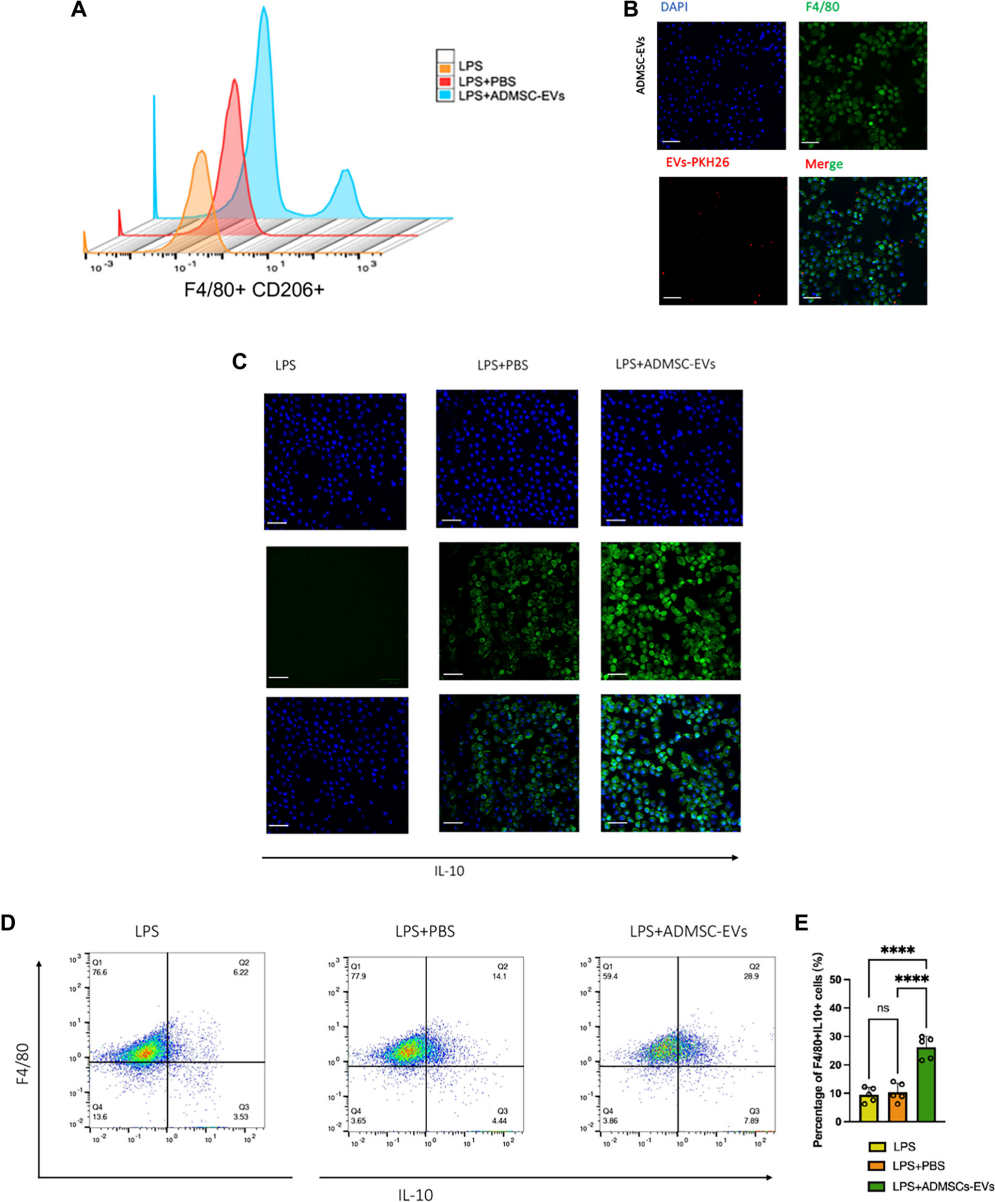
**ADMSC-EVs induce the IL-10 secretion in macrophage *in vivo***. (A) PKH-67-labeled ADMSC-EVs were encapsulated within F4/80-positive macrophages. The co-localization of EVs (PKH67-labeled green) and macrophages (F4/80, red) in lung tissue was assessed using immunofluorescence staining; (B) The percentage of F4/80+ CD206+ macrophages in lung tissue from mice was measured following ADMSC-EV treatment; (C) The expression of IL-10 in lung tissue was evaluated via immunofluorescence staining; (D) The expression of IL-10 in F4/80+ lung macrophages from sepsis mice treated with ADMSC-EVs was determined. Abbreviations: ADMSC-EVs: Adipose-derived mesenchymal stem cell-derived extracellular vesicles; PKH-67: Lipophilic fluorescent membrane dye; EVs: Extracellular vesicles.

To investigate the influence of ADMSC-EVs on IL-10 secretion from M2 macrophages, we analyzed the macrophage uptake of the M2 phenotype *in vivo*. We first isolated macrophages from the lungs of mice and then assessed the percentage of M2 phenotype macrophages. As illustrated in Figure 5A and 5B, ADMSC-EV treatment increased the proportion of M2 macrophages in the lungs compared to controls. Additionally, confocal microscopy demonstrated that F4/80 positive macrophages in the lung effectively uptook PKH-67 labeled ADMSCs-EVs (Figure 5C). Furthermore, flow cytometry and immunofluorescence analyses confirmed that ADMSC-EV treatment elevated IL-10 levels in pulmonary macrophages (Figure 5C and 5D), suggesting a mechanism for reducing pulmonary inflammation through enhanced M2 polarization and IL-10 secretion. We also examined the pathological changes in mice treated with LPS or ADMSC-EVs via HE staining, which indicated that ADMSC-EVs ameliorated sepsis in the lungs (Figure S8). Additionally, we assessed the effects of ADMSC-EVs on various immune cell populations, such as T cells, neutrophils, and dendritic cells, which showed that ADMSC-EVs increased CCR7 expression in dendritic cells while decreasing CCR2 expression in neutrophils (Figure S9).

### Biodistribution of ADMSC-EVs in LPS-induced septic mice

**Figure 6.**
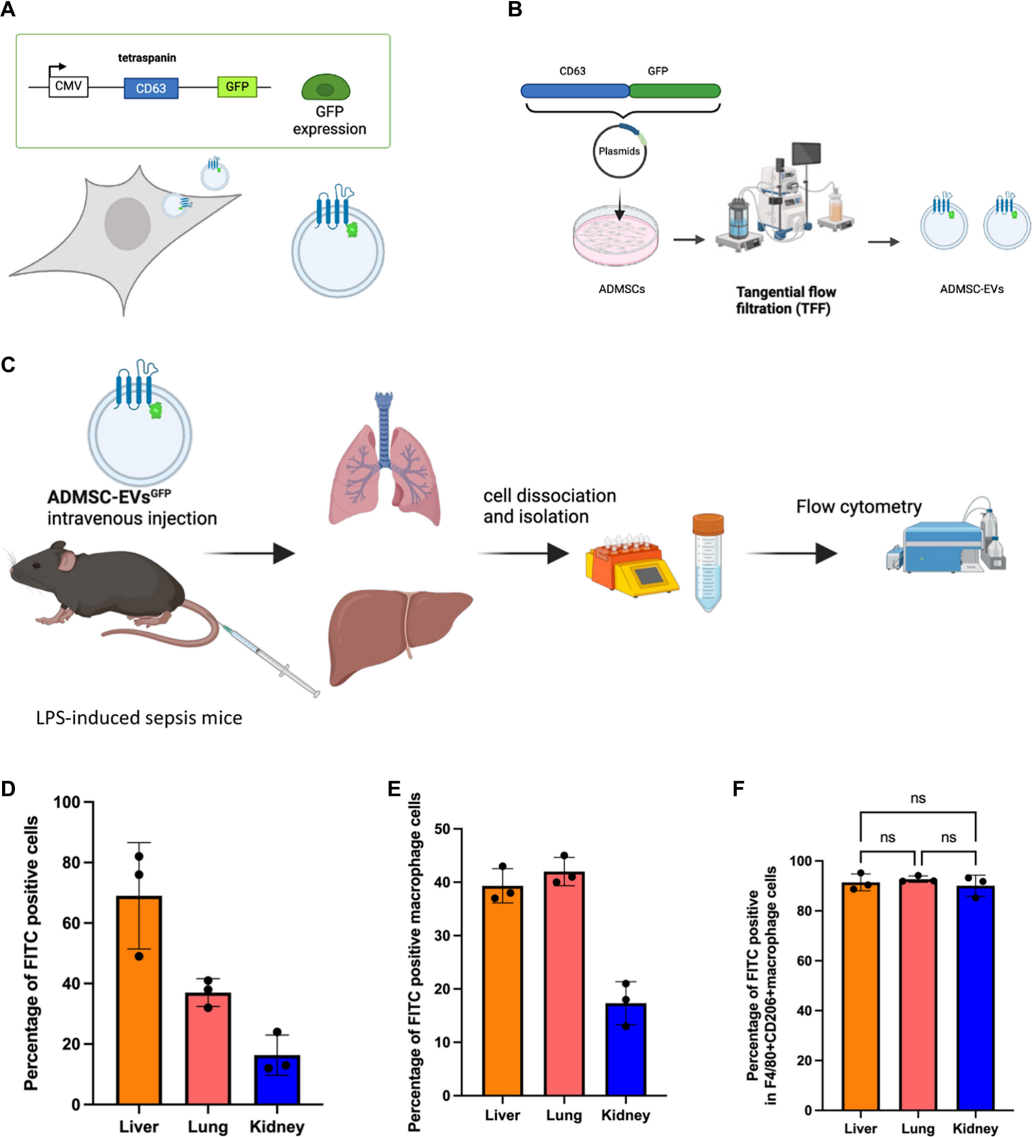
***In vivo* biodistribution of ADMSC-EVs.** (A) Flowchart of plasmid “Plex-CD63-GFP” for engineered ADMSC-EVsGFP; (B) Flowchart of biogenesis process for producing ADMSC-EVsGFP; (C) Flowchart detailing the experimental setup for administering ADMSC-EVsGFP to septic mice to assess their biodistribution *in vivo*; (D) Flow cytometric analysis revealing the percentage of FITC+ cells in the liver, lung, and kidney of septic mice; (E) Flow cytometric analysis indicating the percentage of FITC+ macrophages in the liver, lung, and kidney of septic mice; (F) Flow cytometric analysis showing the percentage of FITC+ M2 macrophages in the liver, lung, and kidney of septic mice. Abbreviations: ADMSC-EVsGFP: Green fluorescent protein-labeled adipose-derived mesenchymal stem cell-derived extracellular vesicles; GFP: Green fluorescent protein; FITC: Fluorescein isothiocyanate; ADMSC-EVs: Adipose-derived mesenchymal stem cell-derived extracellular vesicles.

We assessed the biodistribution of ADMSC-EVs in a model of sepsis induced by LPS. A plasmid encoding the fused protein CD63-GFP (Figure 6A) was transfected into ADMSCs to facilitate *in vivo* tracking of the EVs (Figure 6B). As shown in Figure 6C, ADMSC-EVsGFP were injected into LPS-induced septic mice to evaluate their biodistribution. Figure 6D illustrates that ADMSC-EVsGFP successfully entered and were encapsulated within the lungs, liver, and kidneys of septic mice (Figure 6E). Furthermore, a significant proportion of M2 macrophages in these organs tested positive for green fluorescent protein (GFP), indicating successful induction and reprogramming by ADMSC-EVs. Collectively, these findings suggest that ADMSC-EVs can enter the liver, lung, and kidney, playing critical roles in macrophage polarization.

### ADMSCs-EVs encapsulated miR-21-5p targets *PELI1* in macrophages

**Figure 7.**
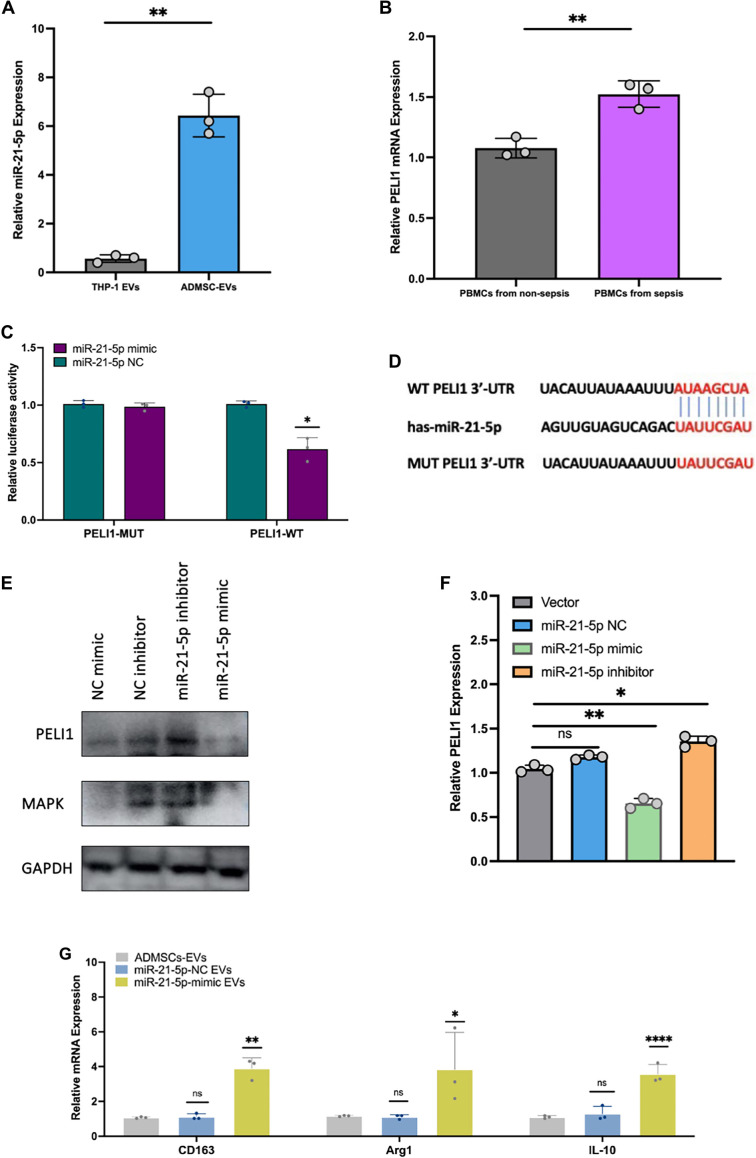
**ADMSC-EVs encapsulated miR-21-5p targeting *PELI1* in macrophages.** (A) Expression levels of miR-21-5p in THP-1 EVs and ADMSC-EVs; (B) mRNA expression levels of *PELI1* in PBMCs from sepsis and non-sepsis patients; (C) Evaluation of the interaction between miR-21-5p and *PELI1* using a dual-luciferase reporter assay; (D) Analysis of the binding site of has-miR-21-5p (the mature form of human miR-21 derived from the 5’ arm of its precursor hairpin structure) and *PELI1* using TargetScan software; (E) Protein expression levels of PELI1 following regulation of miR-21-5p expression; (F) mRNA expression levels of *PELI1* after regulation of miR-21-5p expression; (G) mRNA expression levels of *CD163*, *Arg1*, and *IL-10* following regulation of miR-21-5p expression. Abbreviations: ADMSC-EVs: Adipose-derived mesenchymal stem cell-derived extracellular vesicles; EVs: Extracellular vesicles; PBMCs: Peripheral blood mononuclear cells; *PELI1*: Pellino E3 ubiquitin protein ligase 1; *Arg1*: Arginase 1.

It has been widely reported that decreased expression of *PELI1* in macrophages is associated with the inhibition of inflammation [38]. The expression of miR-21-5p in ADMSC-EVs was elevated compared to that in THP-1 EVs (Figure 7A). In the current study, we found that the expression level of *PELI1* in PBMCs from sepsis patients was significantly higher than that in PBMCs from healthy individuals (Figure 7B). Dual-luciferase assays confirmed that miR-21-5p binding to *PELI1* resulted in decreased luciferase activity (Figure 7C). Mechanistically, we analyzed potential target genes of miR-21-5p using TargetScan. As depicted in Figure 7D, *PELI1* was predicted to be a target gene of miR-21-5p. Furthermore, upregulation of miR-21-5p led to reduced levels of PELI1 and MAPK in THP-1 cells (Figure 7E and 7F and Figures S8 and S11). These findings indicate that ADMSC-EVs encapsulated miR-21-5p can target PELI1 in macrophages, promoting M2 macrophage polarization during sepsis.

## Discussion

Sepsis is recognized as a syndrome characterized by organ dysfunction due to the dysregulation of the host’s response to systemic infection. It remains one of the most challenging and life-threatening conditions in modern medicine, with no effective treatment available beyond supportive care. As a leading cause of death among critically ill patients, sepsis presents a significant global healthcare challenge [39, 40]. Increasing evidence suggests that sepsis triggers a robust innate immune response, primarily mediated by macrophages. These heterogeneous immune cells can be broadly categorized into classically activated M1 macrophages and alternatively activated M2 macrophages, which exhibit pro-inflammatory and anti-inflammatory properties, respectively [41, 42]. M1 macrophages are primarily involved in pro-inflammatory processes; hyperactivated M1 macrophages are a major source of pro-inflammatory cytokines, such as IL-6 and TNF-α, which contribute to tissue damage during infections [43]. In contrast, M2 macrophages play crucial roles in tissue repair and the resolution of inflammation [44]. For example, studies have shown that the transplantation of M2 macrophages can reduce the production of pro-inflammatory cytokines (e.g., IL-6 and TNF-α) in animal models of acute kidney injury and colitis [45, 46].

Stem cells have garnered significant interest for clinical applications due to their differentiation potential [47]. ADMSCs have emerged as a promising alternative for cell-based therapy, offering advantages such as ease of access, ethical acceptance, and abundant availability [48, 49]. ADMSC-based therapies have demonstrated potential to modulate inflammation, promote tissue regeneration, and improve prognosis in various conditions [50, 51]. Consequently, we selected ADMSC-derived EVs as nanocarriers for sepsis treatment. Most of the EVs in our study ranged from 30 to 200 nm, with only a small fraction larger than 200 nm. However, we classified these larger particles as EVs, recognizing the difficulty in isolating specific subpopulations in our study. It is important to note that particles larger than 200 nm should be categorized as microvesicles.

The immunofluorescence images in Figure 2B and 2C display cytoplasmic signals for CD105 and CD90. This is likely attributable to the permeabilization step applied during the staining procedure, which may have partially affected membrane integrity. Nevertheless, the overall expression patterns are consistent with the expected surface marker profiles, as confirmed by flow cytometry (Figure 2D and 2E), which assesses surface marker expression without permeabilization.

EVs are nanosized particles (∼30–1000 nm) secreted by diverse types of cells [52, 53]. EVs retain bioactive properties similar to those of the donor cells, owing to the diverse biomolecules (e.g., proteins [54], nucleic acids [55], lipids, etc.) incorporated during the biogenesis process [56]. Furthermore, EVs retain their bioactivity even after prolonged storage and post-modification [57]. Our previous study demonstrated that the miR-21-5p is highly expressed in MSC derived EVs, and it has been reported that miR-21-5p plays a role in macrophages polarization [58, 59]. Therefore, it is logical to investigate the mechanisms of EVs (particularly those containing miR-21-5p) in influencing macrophages polarization. In addition, it is indeed possible for murine-derived miRNAs to regulate mRNA expression in human cells, despite the species difference. This regulatory effect is primarily based on the sequence complementarity and the conservation of target mRNA sequences across species. Our results are consistence to previous published studies [60, 61], which underscore the potential for murine miRNAs to regulate mRNA expression in human cells based on the conservation of miRNA-target interactions across species. Additionally, scaffold sorting proteins play a crucial role in EVs biogenesis. According to this mechanism, it is possible to engineer bioengineered EVs with diverse cargoes, including GFP proteins. Based on this approach, we generated the ADMSC-EVsGFP to track the distribution of EVs *in vivo*.

In this study, we first evaluated macrophage phenotypes in PBMCs from septic patients and assessed IL-10 cytokine levels in the serum of these patients. The results indicated a lower percentage of M2 macrophages in septic patients’ PBMCs compared to non-septic patients, alongside a reduced IL-10 cytokine level in the serum of septic patients. These results are consistent with the results in other studies [62–65]. Subsequently, we isolated and characterized ADMSCs and ADMSC-EVs using flow cytometry, TEM, NTA, and Western blot assays [10, 66]. We found that ADMSC-EVs could enter macrophages as recipient cells, consistent with previous studies, and induce M2 phenotype *polarization in vitro* and *ex vivo*. These results were validated through flow cytometry, qPCR, and Western blot assays. Furthermore, in animal experiments, we observed that ADMSC-EVs could mitigate LPS-induced sepsis and improve prognosis by inducing M2 polarization *in vivo*. Additionally, we utilized engineered EVs containing GFP to assess the distribution of ADMSC-EVs in sepsis-induced mice, producing a plasmid named “CD63-GFP” to generate ADMSC-EVsGFP, consistent with prior research. Flow cytometry results revealed that most M2 macrophages were GFP-positive, indicating that cells encapsulating ADMSC-EVs polarized into the M2 phenotype. This intricate method provides evidence supporting the role of ADMSC-EVs in polarizing M2 macrophages *in vivo*, with potential applications in future studies. In this context, “F4/80^+^CD206^+^” denotes mouse primary monocytes, while “CD68^+^CD206^+^” corresponds to human THP-1 derived macrophages, reflecting distinct marker profiles for murine and human macrophages.

*In vivo* biodistribution studies of ADMSC-EVs were conducted to evaluate their tissue targeting profiles and address concerns regarding potential off-target effects on non-immune cells. Analysis of PBMCs revealed that ADMSC-EVs increased CCR7 expression in dendritic cells while decreasing CCR2 expression in neutrophils, suggesting their potential therapeutic effects. Our findings indicate that although ADMSC-EVs localized to the lungs, a significant fraction was also detected in the liver, highlighting the need for further optimization to enhance specificity and minimize unintended interactions.

PELI1 is a protein that plays a crucial role in regulating immune responses and inflammation [67]. As a member of the PELI family of proteins, PELI1 is characterized by its peloton-like domains and is particularly implicated in modulating the NF-κB signaling pathway, a key regulator of immune and inflammatory responses. Its functions are closely associated with the regulation of macrophage polarization, specifically the differentiation between pro-inflammatory (M1) and anti-inflammatory (M2) phenotypes. The downstream effects of PELI1 inhibition on M2 macrophage polarization warrant further investigation to determine if other mediators or signaling pathways are involved [68].

*PELI1* (Pel-like 1) serves as a critical regulator of immune responses, and its inhibition could potentially influence multiple signaling cascades central to macrophage polarization, particularly the NF-κB, JAK-STAT, and PI3K-AKT pathways. Additionally, cytokines such as IL-4, IL-13, and TGF-β, which are key drivers of M2 macrophage polarization, may be regulated through PELI1 inhibition. These cytokines activate various transcription factors that promote M2 macrophage characteristics. Consequently, the inhibition of PELI1 could disrupt the signaling pathways initiated by these cytokines, leading to alterations in the polarization process.

Understanding how PELI1 interacts with these signaling pathways and mediators is essential for elucidating the broader molecular mechanisms underlying M2 macrophage polarization. This knowledge could provide valuable insights into the therapeutic potential of targeting PELI1 in diseases where macrophage polarization plays a critical role, such as chronic inflammation, tissue repair, and autoimmune diseases. We hypothesize that elevated levels of PELI1 reflect upstream activation driving uncontrolled inflammation. Therefore, increased *PELI1* expression in septic PBMCs suggests that therapeutics aimed at inhibiting PELI1 could mitigate inflammation.

Regarding the use of PKH-67-labeled ADMSC-EVs in Figures 4 and 5 and ADMSC-EVs^GFP^ in Figure 6, we would like to clarify the rationale behind these different labeling strategies. PKH-67 is a lipid-soluble dye commonly used for cell and vesicle tracking; however, it may exhibit reduced fluorescence intensity over time due to potential dye dilution or degradation during *in vivo* circulation. To address this limitation, we employed ADMSC-EVs^GFP^ in Figure 6, as GFP is a fluorescent protein that remains intact and stable *in vivo*. It is widely reported that CD63-GFP EVs are used to trace the EVs distribution [16, 69], the CD63 is scaffold protein of EVs, and does not influence the EVs distribution.

Nevertheless, several limitations exist in the present study. First, a larger cohort of patients should be enrolled to further explore the relationship between the percentage of M2 macrophages and the severity of sepsis. Second, ADMSC-EVs should be tested in comparison with or in combination with current sepsis treatments to evaluate both the efficacy of individual treatments and potential combination therapies. While additional controls, such as unlabeled ADMSC-EVs, PKH-67 dye alone, and control EVs (e.g., THP-1 EVs or miR-21-5p knockdown/overexpression variants), would be ideal, we believe our experimental design adequately addresses the primary research question without necessitating these controls. Future studies should include seed-mismatch controls or rescue experiments, as well as investigations into EV-borne miR-21 entry and load dependence. Specifically, our *in vitro* experimental results could validate relevant baseline controls in our experimental setup to ensure the LPS-induced sepsis model was appropriately validated. These controls are deemed sufficient to assess the effects of ADMSC-EVs in the context of sepsis.

Another concern is the sample size in the survival assay, where the decision to use *n* ═ 5 per group was based on preliminary data and resource availability considerations. While small sample sizes are acknowledged as a limitation, this study was designed to explore preliminary trends and observe initial efficacy signals. We recognize that smaller group sizes may introduce variability in survival data, particularly in high-lethality models, where a single survivor could disproportionately affect the interpretation of statistical significance. However, we believe this sample size provides valuable insights into the potential efficacy of the treatment under investigation. Furthermore, although we included a balanced mix of sexes (2 males and 3 females per group), we did not perform sex-stratified analyses due to the preliminary nature of the study and limitations in sample size. We understand the importance of exploring potential sex differences in outcomes and plan to address this in future studies with larger sample sizes and more targeted analyses.

In this study, we investigated the therapeutic potential of ADMSC-EVs for sepsis, emphasizing their capacity to modulate macrophage polarization and improve patient outcomes. Our findings reveal a significant reduction in the proportion of M2 macrophages and a decrease in IL-10 cytokine levels in PBMCs of septic patients compared to non-septic controls. These results underscore the disruption of the anti-inflammatory response in sepsis, which contributes to disease progression.

We further demonstrated that ADMSC-EVs effectively promote macrophage polarization to the M2 phenotype both *in vitro* and *in vivo*, as evidenced by flow cytometry, qPCR, and Western blot analysis. Notably, the use of engineered ADMSC-EVs containing GFP allowed us to trace the distribution of these vesicles *in vivo*, confirming their ability to reach macrophages and induce M2 polarization in a LPS-induced sepsis mouse model. These results support the therapeutic potential of ADMSC-EVs in modulating the immune response in sepsis, particularly through the polarization of macrophages to the M2 phenotype, which is essential for tissue repair and resolution of inflammation.

Additionally, we explored the role of PELI1 in regulating macrophage polarization and its potential as a therapeutic target. Inhibition of PELI1 appears to influence key signaling pathways central to macrophage polarization, providing valuable insights into the mechanisms through which ADMSC-EVs exert their effects. Despite the promising results, this study has limitations, including the necessity for a larger patient cohort and further evaluation of ADMSC-EVs in conjunction with existing sepsis treatments. Nevertheless, our findings lay the groundwork for future research aimed at optimizing ADMSC-EV-based therapies and investigating their clinical applicability in sepsis and other inflammatory conditions.

## Conclusion

In conclusion, our study demonstrates that ADMSC-EVs can reprogram macrophages into the M2 phenotype and mitigate LPS-mediated sepsis *in vivo*. Mechanistically, ADMSC-EVs deliver miR-21-5p to target PELI1, thereby promoting M2 macrophage polarization and enhancing IL-10 secretion. These findings offer promising insights into the therapeutic potential of ADMSC-EVs as a novel strategy for sepsis treatment.

**Consent to participate:** Freely given, informed consent to participate in the study was obtained from all participants (or their parent or legal guardian in the case of children under 16). The tissues were collected in Zhongshan Hospital (Shanghai, China) and Fudan University. No tissues were obtained from prisoners.

## Supplemental data

Supplemental data are available at the following link: https://www.bjbms.org/ojs/index.php/bjbms/article/view/11971/4030.

## Data Availability

The datasets used and analyzed during the current study are available from the corresponding author on reasonable request.

## References

[1] Cecconi M, Evans L, Levy M, Rhodes A (2018). Sepsis and septic shock. Lancet.

[2] Angus DC, van der Poll T (2013). Severe sepsis and septic shock. N Engl J Med.

[3] Seymour CW, Liu VX, Iwashyna TJ, Brunkhorst FM, Rea TD, Scherag A (2016). Assessment of clinical criteria for sepsis: for the third international consensus definitions for sepsis and septic shock (sepsis-3). JAMA.

[4] Paugam-Burtz C, Levesque E, Louvet A, Thabut D, Amathieu R, Bureau C (2020). Management of liver failure in general intensive care unit. Anaesth Crit Care Pain Med.

[5] Legese MH, Asrat D, Swedberg G, Hasan B, Mekasha A, Getahun T (2022). Sepsis: emerging pathogens and antimicrobial resistance in Ethiopian referral hospitals. Antimicrob Resist Infect Control.

[6] Chaurasia S, Sivanandan S, Agarwal R, Ellis S, Sharland M, Sankar MJ (2019). Neonatal sepsis in South Asia: huge burden and spiralling antimicrobial resistance. BMJ.

[7] Fang F, Zhang Y, Tang J, Lunsford LD, Li T, Tang R (2019). Association of corticosteroid treatment with outcomes in adult patients with sepsis: a systematic review and meta-analysis. JAMA Intern Med.

[8] Uccelli A, Moretta L, Pistoia V (2008). Mesenchymal stem cells in health and disease. Nat Rev Immunol.

[9] Hu C, Li L (2018). Preconditioning influences mesenchymal stem cell properties in vitro and in vivo. J Cell Mol Med.

[10] Xia L, Zhang C, Lv N, Liang Z, Ma T, Cheng H (2022). AdMSC-derived exosomes alleviate acute lung injury via transferring mitochondrial component to improve homeostasis of alveolar macrophages. Theranostics.

[11] Hu C, Zhao L, Li L (2019). Current understanding of adipose-derived mesenchymal stem cell-based therapies in liver diseases. Stem Cell Res Ther.

[12] Abreu SC, Antunes MA, Xisto DG, Cruz FF, Branco VC, Bandeira E (2017). Bone marrow, adipose, and lung tissue-derived murine mesenchymal stromal cells release different mediators and differentially affect airway and lung parenchyma in experimental asthma. Stem Cells Transl Med.

[13] Zhu Z, Zhang Y, Zhang Y, Zhang H, Liu W, Zhang N (2019). Exosomes derived from human umbilical cord mesenchymal stem cells accelerate growth of VK2 vaginal epithelial cells through MicroRNAs in vitro. Hum Reprod.

[14] Zhang H, Freitas D, Kim HS, Fabijanic K, Li Z, Chen H (2018). Identification of distinct nanoparticles and subsets of extracellular vesicles by asymmetric flow field-flow fractionation. Nat Cell Biol.

[15] Gu Y, Zhou G, Zhang M, Lu G, Shen F, Qi B (2025). Bioengineered extracellular vesicles presenting PD-L1 and Gal-9 to ameliorate new-onset primary ovarian insufficiency (POI). Chem Eng J.

[16] Zhou G, Gu Y, Zhang M, Ding J, Lu G, Hua K (2025). Identification of genetically engineered strategies to manipulate nano-platforms presenting immunotherapeutic ligands for alleviating primary ovarian insufficiency progression. Cell Commun Signal.

[17] Gao X, Ran N, Dong X, Zuo B, Yang R, Zhou Q (2018). Anchor peptide captures, targets, and loads exosomes of diverse origins for diagnostics and therapy. Sci Transl Med.

[18] Alvarez-Erviti L, Seow Y, Yin H, Betts C, Lakhal S, Wood MJ (2011). Delivery of siRNA to the mouse brain by systemic injection of targeted exosomes. Nat Biotechnol.

[19] Han G, Zhang Y, Zhong L, Wang B, Qiu S, Song J (2024). Generalizable anchor aptamer strategy for loading nucleic acid therapeutics on exosomes. EMBO Mol Med.

[20] Saad N, Duroux-Richard I, Touitou I, Jeziorski E, Apparailly F (2023). MicroRNAs in inflammasomopathies. Immunol Lett.

[21] Schütte JP, Manke MC, Hemmen K, Münzer P, Schörg BF, Ramos GC (2023). Platelet-derived MicroRNAs regulate cardiac remodeling after myocardial ischemia. Circ Res.

[22] Zeng H, Zhou Y, Liu Z, Liu W (2024). MiR-21-5p modulates LPS-induced acute injury in alveolar epithelial cells by targeting SLC16A10. Sci Rep.

[23] Xue J, Liu J, Xu B, Yu J, Zhang A, Qin L (2021). miR-21-5p inhibits inflammation injuries in LPS-treated H9c2 cells by regulating PDCD4. Am J Transl Res.

[24] Weber B, Henrich D, Marzi I, Leppik L (2024). Decrease of exosomal miR-21-5p and the increase of CD62p+ exosomes are associated with the development of sepsis in polytraumatized patients. Mol Cell Probes.

[25] Zhang Y, Huang H, Liu W, Liu S, Wang XY, Diao ZL (2021). Endothelial progenitor cells-derived exosomal microRNA-21-5p alleviates sepsis-induced acute kidney injury by inhibiting RUNX1 expression. Cell Death Dis.

[26] Watson DC, Johnson S, Santos A, Yin M, Bayik D, Lathia JD (2021). Scalable isolation and purification of extracellular vesicles from escherichia coli and other bacteria. J Vis Exp.

[27] Welsh JA, Goberdhan DCI, O’Driscoll L, Buzas EI, Blenkiron C, Bussolati B (2024). Minimal information for studies of extracellular vesicles (MISEV2023): from basic to advanced approaches. J Extracell Vesicles.

[28] Welsh JA, Arkesteijn GJA, Bremer M, Cimorelli M, Dignat-George F, Giebel B (2023). A compendium of single extracellular vesicle flow cytometry. J Extracell Vesicles.

[29] Welsh JA, Van Der Pol E, Arkesteijn GJA, Bremer M, Brisson A, Coumans F (2020). MIFlowCyt-EV: a framework for standardized reporting of extracellular vesicle flow cytometry experiments. J Extracell Vesicles.

[30] Zhang L, Jiao G, Ren S, Zhang X, Li C, Wu W (2020). Exosomes from bone marrow mesenchymal stem cells enhance fracture healing through the promotion of osteogenesis and angiogenesis in a rat model of nonunion. Stem Cell Res Ther.

[31] Yu L, Sui B, Fan W, Lei L, Zhou L, Yang L (2021). Exosomes derived from osteogenic tumor activate osteoclast differentiation and concurrently inhibit osteogenesis by transferring COL1A1-targeting miRNA-92a-1-5p. J Extracell Vesicles.

[32] Li B, Xia C, He W, Liu J, Duan R, Ji Z (2024). The thyroid hormone analog GC-1 mitigates acute lung injury by inhibiting M1 macrophage polarization. Adv Sci (Weinh).

[33] Zhou G, Zhou F, Gu Y, Zhang M, Zhang G, Shen F (2022). Vaginal microbial environment skews macrophage polarization and contributes to cervical cancer development. J Immunol Res.

[34] Théry C, Witwer KW, Aikawa E, Alcaraz MJ, Anderson JD, Andriantsitohaina R (2018). Minimal information for studies of extracellular vesicles 2018 (MISEV2018): a position statement of the International Society for Extracellular Vesicles and update of the MISEV2014 guidelines. J Extracell Vesicles.

[35] Witwer KW, Goberdhan DC, O’Driscoll L, Théry C, Welsh JA, Blenkiron C (2021). Updating MISEV: evolving the minimal requirements for studies of extracellular vesicles. J Extracell Vesicles.

[36] Li Y, Tang X, Gu Y, Zhou G (2023). Adipocyte-derived extracellular vesicles: small vesicles with big impact. Front Biosci (Landmark Ed).

[37] Théry C, Amigorena S, Raposo G, Clayton A (2006). Isolation and characterization of exosomes from cell culture supernatants and biological fluids. Curr Protoc Cell Biol.

[38] Thirunavukkarasu M, Swaminathan S, Kemerley A, Pradeep SR, Lim ST, Accorsi D (2023). Role of pellino-1 in inflammation and cardioprotection following severe sepsis: a novel mechanism in a murine severe sepsis model (†). Cells.

[39] Cox MI, Voss H (2021). Improving sepsis recognition and management. Curr Probl Pediatr Adolesc Health Care.

[40] Rello J, Valenzuela-Sánchez F, Ruiz-Rodriguez M, Moyano S (2017). Sepsis: a review of advances in management. Adv Ther.

[41] Yang K, Fan M, Wang X, Xu J, Wang Y, Tu F (2022). Lactate promotes macrophage HMGB1 lactylation, acetylation, and exosomal release in polymicrobial sepsis. Cell Death Differ.

[42] Patoli D, Mignotte F, Deckert V, Dusuel A, Dumont A, Rieu A (2020). Inhibition of mitophagy drives macrophage activation and antibacterial defense during sepsis. J Clin Invest.

[43] Shapouri-Moghaddam A, Mohammadian S, Vazini H, Taghadosi M, Esmaeili SA, Mardani F (2018). Macrophage plasticity, polarization, and function in health and disease. J Cell Physiol.

[44] Atri C, Guerfali F, Laouini D (2018). Role of human macrophage polarization in inflammation during infectious diseases. Int J Mol Sci.

[45] Li Z, Xiao J, Xu X, Li W, Zhong R, Qi L (2021). M-CSF, IL-6, and TGF-ß promote generation of a new subset of tissue repair macrophage for traumatic brain injury recovery. Sci Adv.

[46] Bouchareychas L, Duong P, Covarrubias S, Alsop E, Phu TA, Chung A (2020). Macrophage exosomes resolve atherosclerosis by regulating hematopoiesis and inflammation via MicroRNA cargo. Cell Rep.

[47] Pajarinen J, Lin T, Gibon E, Kohno Y, Maruyama M, Nathan K (2019). Mesenchymal stem cell-macrophage crosstalk and bone healing. Biomaterials.

[48] Tobita M, Orbay H, Mizuno H (2011). Adipose-derived stem cells: current findings and future perspectives. Discov Med.

[49] Payab M, Goodarzi P, Foroughi Heravani N, Hadavandkhani M, Zarei Z, Falahzadeh K (2018). Stem cell and obesity: current state and future perspective. Adv Exp Med Biol.

[50] Zheng G, Huang L, Tong H, Shu Q, Hu Y, Ge M (2014). Treatment of acute respiratory distress syndrome with allogeneic adipose-derived mesenchymal stem cells: a randomized, placebo-controlled pilot study. Respir Res.

[51] Sun J, Ding X, Liu S, Duan X, Liang H, Sun T (2020). Adipose-derived mesenchymal stem cells attenuate acute lung injury and improve the gut microbiota in septic rats. Stem Cell Res Ther.

[52] van Niel G, D’Angelo G, Raposo G (2018). Shedding light on the cell biology of extracellular vesicles. Nat Rev Mol Cell Biol.

[53] Wang G, Li J, Bojmar L, Chen H, Li Z, Tobias GC (2023). Tumour extracellular vesicles and particles induce liver metabolic dysfunction. Nature.

[54] Li Z, Low V, Luga V, Sun J, Earlie E, Parang B (2022). Tumor-produced and aging-associated oncometabolite methylmalonic acid promotes cancer-associated fibroblast activation to drive metastatic progression. Nat Commun.

[55] Abels ER, Breakefield XO (2016). Introduction to extracellular vesicles: biogenesis, RNA cargo selection, content, release, and uptake. Cell Mol Neurobiol.

[56] Kenific CM, Zhang H, Lyden D (2021). An exosome pathway without an ESCRT. Cell Res.

[57] Choi H, Kim Y, Mirzaaghasi A, Heo J, Kim YN, Shin JH (2020). Exosome-based delivery of super-repressor IκBα relieves sepsis-associated organ damage and mortality. Sci Adv.

[58] Qian W, Wu E, Chen H, Yao J, Wang J, Zhou Y (2024). MSCs-exosomes can promote macrophage M2 polarization via exosomal miR-21-5p through mesenteric injection: a promising way to attenuate murine colitis. J Crohns Colitis.

[59] Hu Z, You L, Hu S, Yu L, Gao Y, Li L (2024). Hepatocellular carcinoma cell-derived exosomal miR-21-5p promotes the polarization of tumor-related macrophages (TAMs) through SP1/XBP1 and affects the progression of hepatocellular carcinoma. Int Immunopharmacol.

[60] Agarwal V, Bell GW, Nam JW, Bartel DP (2015). Predicting effective microRNA target sites in mammalian mRNAs. Elife.

[61] Lu J, Getz G, Miska EA, Alvarez-Saavedra E, Lamb J, Peck D (2005). MicroRNA expression profiles classify human cancers. Nature.

[62] Jain K, Mohan KV, Roy G, Sinha P, Jayaraman V, Kiran F (2023). Reconditioned monocytes are immunomodulatory and regulate inflammatory environment in sepsis. Sci Rep.

[63] Xie Z, Lin B, Jia X, Su T, Wei Y, Tang J (2021). Enhanced IL-10 inhibits proliferation and promotes apoptosis of HUVECs through STAT3 signaling pathway in sepsis. Histol Histopathol.

[64] Fan L, Yao L, Li Z, Wan Z, Sun W, Qiu S (2023). Exosome-based mitochondrial delivery of circRNA mSCAR alleviates sepsis by orchestrating macrophage activation. Adv Sci (Weinh).

[65] Chen X, Liu Y, Gao Y, Shou S, Chai Y (2021). The roles of macrophage polarization in the host immune response to sepsis. Int Immunopharmacol.

[66] Kuang Y, Zheng X, Zhang L, Ai X, Venkataramani V, Kilic E (2020). Adipose-derived mesenchymal stem cells reduce autophagy in stroke mice by extracellular vesicle transfer of miR-25. J Extracell Vesicles.

[67] Hwang S, Park J, Koo SY, Lee SY, Jo Y, Ryu D (2025). The ubiquitin ligase Pellino1 targets STAT3 to regulate macrophage-mediated inflammation and tumor development. Nat Commun.

[68] Dai D, Zhou H, Yin L, Ye F, Yuan X, You T (2022). PELI1 promotes radiotherapy sensitivity by inhibiting noncanonical NF-κB in esophageal squamous cancer. Mol Oncol.

[69] Silva AM, Lázaro-Ibáñez E, Gunnarsson A, Dhande A, Daaboul G, Peacock B (2021). Quantification of protein cargo loading into engineered extracellular vesicles at single-vesicle and single-molecule resolution. J Extracell Vesicles.

